# Transcription factor specificity limits the number of DNA-binding motifs

**DOI:** 10.1371/journal.pone.0263307

**Published:** 2022-01-28

**Authors:** Ariel A. Aptekmann, Denys Bulavka, Alejandro D. Nadra, Ignacio E. Sánchez

**Affiliations:** 1 Facultad de Ciencias Exactas y Naturales, Laboratorio de Fisiología de Proteínas, Consejo Nacional de lnvestigaciones Cientificas y Técnicas, Instituto de Química Biológica de la Facultad de Ciencias Exactas y Naturales (IQUIBICEN), Universidad de Buenos Aires, Buenos Aires, Argentina; 2 Marine and Coastal Sciences Department, Rutgers University, New Brunswick, New Jersey, United States of America; 3 Departamento de Matematica, Facultad de Ciencias Exactas y Naturales, Universidad de Buenos Aires, Buenos Aires, Argentina; 4 Facultad de Ciencias Exactas y Naturales, Departamento de Fisiología, Biología Molecular y Celular, IB3, Universidad de Buenos Aires, Buenos Aires, Argentina; Southern Illinois University, UNITED STATES

## Abstract

We study the limits imposed by transcription factor specificity on the maximum number of binding motifs that can coexist in a gene regulatory network, using the SwissRegulon Fantom5 collection of 684 human transcription factor binding sites as a model. We describe transcription factor specificity using regular expressions and find that most human transcription factor binding site motifs are separated in sequence space by one to three motif-discriminating positions. We apply theorems based on the pigeonhole principle to calculate the maximum number of transcription factors that can coexist given this degree of specificity, which is in the order of ten thousand and would fully utilize the space of DNA subsequences. Taking into account an expanded DNA alphabet with modified bases can further raise this limit by several orders of magnitude, at a lower level of sequence space usage. Our results may guide the design of transcription factors at both the molecular and system scale.

## Introduction

In order to understand and preserve molecular biodiversity, it is valuable to investigate if evolution has explored all the options that are possible in theory. In recent years, theoretical limits and empirical estimations for the diversity of protein folds [1], protein families [2], protein-protein interactions [3] and protein linear motifs [4, 5] have been proposed.

Gene networks regulate the expression of up to thousands of genes via interactions between genomic DNA and proteins such as transcription factors [6, 7]. In nature, the components of gene regulatory networks interact in a specific manner: each transcription factor usually recognizes a subset of all possible genomic DNA subsequences and different transcription factors usually recognize non-overlapping sets of DNA subsequences. Some natural transcription factors show similar binding specificities [8]. However, crosstalk between the biological signals read by the hundreds of different transcription factors in a proteome may be detrimental at a cellular scale and may imposes a global constraint on the functioning and evolution of regulatory networks [9]. The specificity of transcription factors is not without consequence. The number of possible sets of genomic DNA subsequences of a given length is finite, regardless of the degree of overlap between the sets. This implies that the number of transcription factors regulating a given gene network through specific recognition of partially- or non-overlapping sets of genomic DNA sequences is finite as well. In other words, the number of binding motifs in a gene regulatory network can only be so large if the sets of DNA subsequences recognized by its transcription factors overlap only so much. This work aims at using empirical measures of transcription factor specificity to calculate a theoretical upper limit for the number of transcription factors that can properly function in the same gene network.

Transcription factor specificity is usually characterized in terms of transcription factor binding sites (TFBS), i.e., the set of DNA subsequences that are recognized by a certain transcription factor. Characterization of TFBS usually starts by the experimental and/or computational identification of several DNA subsequences (termed TFBS instances) that perform a certain function. Once multiple instances of a TFBS are known, a TFBS motif is defined as the set of all TFBS instances that match with a given model (i.e., the set of sites to which a transcription factor binds preferentially) [10]. Most TFBS are short degenerate DNA subsequences of up to 30 base pairs long [11]. The computational definition of the nucleotide pattern for a TFBS motif can be a fixed consensus sequence, a regular expression, or a scoring matrix. This work describes TFBS motifs using regular expressions, which state in a sequential manner which characters are allowed in each position of the motif. For example, in this work we describe the motif for the Arx transcription factor with the ten-character long regular expression [CA][AG][TC][TC]AATT[AG][AG] (S1 Fig). DNA subsequences that are instances of the ARX motif may have a C or an A in the first position of the subsequence, an A or a G in the second position, and so on. Here, we equal the number of coexisting transcription factors to the corresponding number of TFBS motifs.

We focus on human transcription factors as a well studied and relevant example. Current databases report a lower bound for the number of TFBS, since the current set of human transcription factors may not have reached its maximal size. The SwissRegulon Fantom5 collection currently contains annotations for 684 different TFBS motifs in the human genome [12], providing a first empirical lower bound. From a different viewpoint, there are 2604 predicted human protein with DNA-binding domains [7]. If each of these proteins recognizes a different TFBS motif, a second empirical lower bound would be 2604 TFBS motifs in the human genome. Published theoretical estimations from first principles provide upper bounds for the number of coexisting TFBS motifs as a function of motif length and specificity requirements. We may consider as upper bound that there may be as many specific TFBS motifs of length *n* as the maximum number of sequences of length *n*, which is *A*(*n*) = 4^*n*^. This seems unrealistic because most TFBS include multiple instances. A finer theoretical upper bound comes from treating the mapping between transcription factors and binding sequences as a coding problem, where the code words are DNA subsequences of length *n* and the messages are transcription factors [13]. In the limit of large errors, the maximal number of coded messages is bounded by the coloring number of the minimal surface which can embed the code word graph. This provides a second upper bound for the number of minimally overlapping TFBS motifs: $A\left(n\right)∼3.5+\sqrt{0.75\cdot 4^{n}\cdot \left(n\left(4-1\right)-4\right))}$. An alternative approach [14] takes into account motif specificity, measured as the minimal Hamming distance (the minimal number of sequence changes between two instances belonging to different TFBS). The number of TFBS motifs of length *n* with a minimal Hamming distance *d* between sequences belonging to different motifs has a third theoretical upper bound of *A*(*n*, *d*) ≤ 4^*n*−*d*+1^. Thus, a linear increase in transcription factor specificity *d* leads to an exponential decrease in the maximal number of coexisting TFBS motifs *A*. In sum, the effects of both motif length and specificity on the theoretical upper bounds for the maximal number of TFBS motifs are strong.

Published estimations for the maximal number of coexisting TFBS motifs assume a four letter DNA alphabet. However, many genomes harbor up to dozens of different modified bases [15] that are a key aspect of epigenetic regulation. These modified bases include N4-methylcytosine, 5-methylcytosine and 6-methyladenine [16], 5-Hydroxymethylcytosine [17], 5- Formyl and 5-Carboxylcytosine [18] and N6-methyldeoxyadenosine [19]. Modified bases can modulate binding of transcription factors to DNA and thus play a role in TFBS motif encoding. For example, 8-oxo-7,8-dihydroguanine is a signaling agent for gene activation [20] and the presence of 5-methylcytosine can both increase and decrease binding, depending on the transcription factor [21]. We propose that the effective alphabet size of DNA may be over ten letters, which would significantly increase all theoretical estimates for the maximal number of coexisting TFBS motifs.

Maximizing the number of TFBS motifs encoded in a genome should also increase the fraction of all DNA subsequences of length *n* that are an instance of a TFBS motif. In turn, this reduces the number of DNA subsequences that can be used to code exclusively for protein sequences [22] and for other molecular processes involving DNA. To our knowledge, this trade-off between TFBS encoding and the occupancy of DNA sequence space has not been investigated.

Previous work from our group studied the theoretical limits for the number of functional protein motifs [5]. We measured the distance in sequence space for a pair of protein motif classes by quantifying how many motif-discriminating positions prevent a protein subsequence from matching the regular expressions for two classes at once. We derived theorems for the maximal number of motif classes that can simultaneously maintain a certain number of motif-discriminating positions between all pairs of classes in the motif universe, for a given amino acid alphabet. We also calculated the fraction of all protein subsequences that would belong to a motif class if all potential motif classes came into existence. Here, we tackle the question of how many TFBS motifs can potentially coexist in a genome by applying the same theory to empirical data specific for transcription factor binding sites, such as length, specificity and stable base modifications.

## Methods

### Database of transcription factor binding site motifs

All available 684 TFBS weight matrices from the SwissRegulon hg19 database Fantom5 collection [12] were retrieved in June 2018. As expected from the biophysics of protein-DNA interactions [23], TFBS motifs present different levels of sequence conservation at each position. We use the base frequencies *b*_*i*_ as input to convert each TFBS weight matrix from the original database to a regular expression as follows. For each position of the matrix we used the observed frequencies *b*_*i*_ for A, C, G and T to calculate the Effective Alphabet Size (*EAS*) [24]. The *EAS* can be interpreted as the number of equally frequent letters whose Shannon entropy equals the Shannon entropy of the observed frequencies *b*_*i*_ [24]:

$$\begin{array}{c} EAS=2^{-\sum b_{i}\;{\text{log}}_{2}\;b_{i}} \end{array}$$ (1)

Following Shannon´s definition of entropy [24], if a *b*_*i*_ = 0, the corresponding term in *EAS* is zero.

Our model incorporates conservation in a quantitative manner that determines that more conserved (lower entropy) positions will allow less letters than less conserved, (higher entropy) positions. The use of this formula implies that the information content of the regular expression is as close as possible to the information content of the base frequencies used in the calculation. We then assigned *EAS* letters to that position of the regular expression, by order of decreasing frequency. Last, we removed from the regular expression flanking positions that allow for all four bases. Example calculations for the Arx TFBS motif are shown in the S1 Text and the resulting TFBS are included in the S1 File.

### Sequence specificity of transcription factor binding site motifs

We follow previous work [5], in which we used the pigeonhole principle to to calculate the maximal number of coexisting protein linear motifs. Application of this theory to TFBS motifs uses the same formula but accounts for the differences in alphabet size, motif length and motif specificity.

Briefly, the regular expression for a TFBS motif of length *n* can be written as a sequence **A** = (*A*_1_, …, *A*_*n*_) where each *A*_*i*_ is a subset of A={A,C,G,T}. A TFBS motif instance is a sequence (*a*_1_, …, *a*_*n*_) with *a*_*i*_ ∈ *A*_*i*_ for all *i*. We define the structure of **A** as the sequence *e* = (|*A*_1_|, …, |*A*_*n*_|), i.e., the number of allowed bases at each position.

We characterize TFBS specificity using the separation in sequence space between two TFBS regular expressions, measured as the number of motif-discriminating positions. Given an alignment of two TFBS regular expressions **A** = (*A*_1_, …, *A*_*n*_) and **B** = (*B*_1_, …, *B*_*m*_), the number of *motif-discriminating positions* is the number of aligned positions where no letter can match both regular expressions:

$$\begin{array}{c} mdp\text{AB}=|\{i\in \left\{1,\ldots ,n\right\}:A_{i}\cap B_{i}=\emptyset |. \end{array}$$ (2)

If the two TFBS regular expressions **A** and **B** present different lenghts, multiple alignments are possible. We then calculate *mdp* **AB** for all the alignments between the two corresponding regular expressions that do not leave a hanging end for the shorter regular expression and match at least one pair of positions with less than four allowed letters. Finally, we take the minimal *mdp* **AB** across all relevant alignments as a lower limit for the separation in sequence space between the two TFBS motifs.

When the number of TFBS motif-discriminating positions is 0 for a given pair of motifs, we calculate an alternative measure of specificity as 1—(number of sequences that match both regular expressions / number of sequences that match at least one of the regular expressions) (i.e., 1 minus the Jaccard similarity index). A DNA sequence matches a regular expression if all letters in the DNA sequence are allowed by the regular expression. If a letter in one or more positions of a DNA sequence is not allowed by the regular expression, the DNA sequence does not match the regular expression.

### Number of potential transcription factor binding site motifs

For a given TFBS motif structure **e** = (*e*_1_, …, *e*_*n*_) of length *n* and a number *k* of motif-discriminating positions, |M(k)| denotes the maximal number of TFBS motifs satisfying the property that every pair of motifs have at least *k* motif-discriminating positions [5].

|M(0)|≤∏1≤i≤n(3ei-1), (3)

$$\begin{array}{c} \prod_{1\leq i\leq n}\left\lfloor 4/e_{i}\right\rfloor \leq \left|M\left(1\right)\right|\leq \prod_{1\leq i\leq n}4/e_{i}, \end{array}$$ (4)

$$\begin{array}{c} \left|M\left(k<n\right)\right|\leq \prod_{1\leq i\leq n-(k-1)}4/e_{i}\; \end{array}$$ (5)

$$\begin{array}{c} \left|M\left(n\right)\right|=\underset{1\leq i\leq n}{\text{min}}\left\lfloor 4/e_{i}\right\rfloor . \end{array}$$ (6)

Where ⌊*x*⌋ denotes the floor of *x*, i.e. the greatest integer less than or equal to *x*.

The maximal number of TFBS motifs is bounded by inequality. Since we are focused on estimating a theoretical upper limit, all calculations reported in the results section use the upper limit in these formulae. None of the equations in this section are affected by the frequency of bases in the genome, as they deal with the number of different bases allowed on a position, not with which specific base is allowed or its background frequency. Example calculations for the Arx TFBS motif are shown in the S1 Text.

### Occupancy of the sequence space

The fraction of the DNA sequence space occupied by a motif of structure **e** = (*e*_1_, …, *e*_*n*_) is the fraction of all possible DNA subsequences of length *n* that are an instance of the motif:

$$\begin{array}{c} PotentialOccupancy\left(\text{e},k\right)≔\prod_{1\leq i\leq n}\left(e_{i}/4\right) \end{array}$$ (7)

For a set of coexisting TFBS motifs of length *n*, the potential occupancy of sequence space is the fraction of all possible DNA subsequences of length *n* that are an instance of any of the TFBS motifs in the set. In the case of zero motif-discriminating positions, each DNA subsequence may belong to multiple motifs and we were not able to find a formula for the potential occupancy of sequence space [5]. For values of *k* of one or more motif-discriminating positions, motif instances belong to a single motif and the total occupancy of the DNA sequence space is the result from Eq 7 times the number of coexisting motifs, |M(k)|. Example calculations for the Arx TFBS motif are shown in the S1 Text.

## Results

### Sequence specificity of known transcription factor binding site motifs

**Fig 1:**
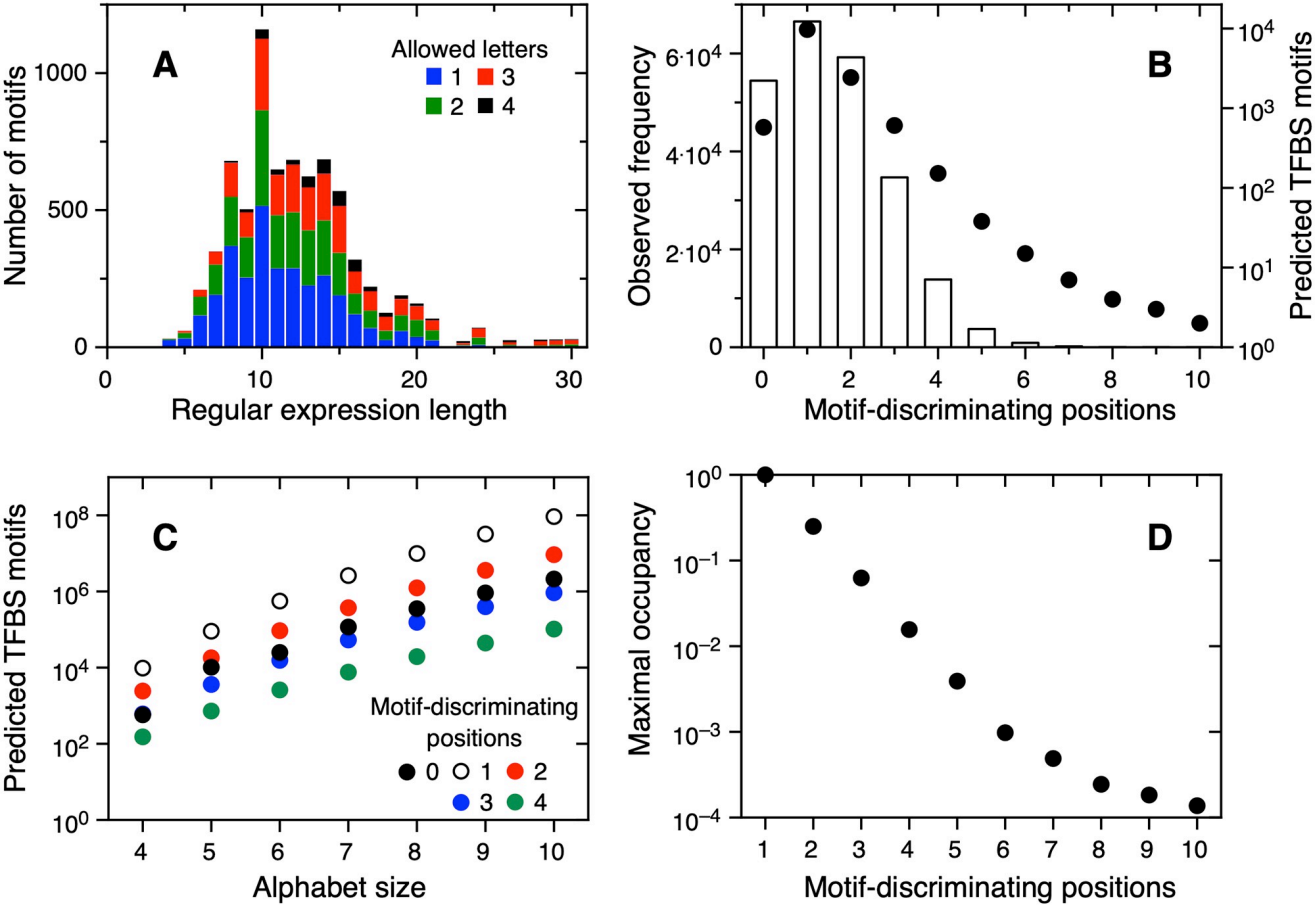
Known and predicted transcription factor binding site motifs. (A) Regular expression length and number of letters allowed for TFBS motifs in the SwissRegulon Fantom5 collection. (B) Bars (left Y axis): Motif-discriminating positions for every pair of TFBS motifs in the SwissRegulon Fantom5 collection. Black circles (right Y axis): Theoretical estimation of the maximal number of coexisting TFBS motifs, as a function of the minimal requirement of motif-discriminating positions. (C) Theoretical estimation of the maximal number of coexisting TFBS motifs, as a function of alphabet size. (D) Potential occupancy of the DNA sequence space by TFBS motifs for an alphabet size of 4 as a function of the number of motif-discriminating positions.

SwissRegulon is a database containing genome-wide annotations of regulatory sites in the intergenic regions of genomes [12]. The regulatory site annotations are produced using a number of recently developed algorithms that operate on multiple alignments of orthologous intergenic regions from related genomes in combination with, whenever available, known sites from the literature, and ChIP-on-chip binding data. We consider positional weight matrices for 684 TFBS motifs in the SwissRegulon Fantom5 collection (section). We generate a regular expression from each matrix, using information theory to minimize the loss of information (section). Fig 1A shows the frequency of each motif length in the database and of the number of symbols allowed at each position. TFBS motif length ranges from 4 to 30 characters. As expected for eukaryotic TFBS motifs, most motifs have lengths between 5 and 20 characters, with a peak at 10 characters.

We quantify the separation in sequence space between a pair of TFBS motifs as the number of motif-discriminating positions (section and S1 Fig). This number is the minimal count of positions where no symbol can match both regular expressions, for every possible alignment where the number of aligned positions is the length of the shorter regular expression [5]. Since other positions might not fully overlap, this is a lower limit for the separation in sequence space between the two TFBS motifs. We calculate the number of motif-discriminating positions for all possible 233586 pairs of TFBS motifs in our database (Fig 1B, white bars and left Y axis). In 77% of the comparisons the two regular expressions are separated in sequence space by at least one motif-discriminating position. This is in agreement with the use of regular expressions, where a mismatch at a single position is enough to rule out that a DNA subsequence belongs to a given TFBS motif. On the other hand, it is rare to find pairs of regular expressions separated by more than five motif-discriminating positions. 23% of regular expressions pairs are not separated in sequence space by a motif-discriminating position. In this case, we measure the separation in sequence space using the fraction of DNA subsequences matching any of the two regular expressions that match only one of them (section). We find that 95% of motif pairs share less than 5% of sequences (S2 Fig). We conclude that SwissRegulon Fantom5 motif pairs show significant separation in sequence space, in agreement with our assumption that there is little cross-talk between natural TFBS motifs.

### Number of potential transcription factor binding site motifs

We use our theory based on the pigeonhole principle (section and [5]) and the structures of TFBS motifs in the SwissRegulon Fantom5 collection (Fig 1A) to estimate the number of SwissRegulon Fantom5-like TFBS motifs that can potentially coexist in nature. We first convert each regular expression in our database to a motif structure (section), which is a vector that quantifies the number of allowed bases at each position. For each structure and a number of motif-discriminating positions, we calculate the number of potential TFBS motifs. As expected from the heterogeneity in motif lengths and structures, the calculated numbers of potential TFBS motifs span several orders of magnitude (S3 Fig). We report the median of the distribution of the number of potential TFBS motifs in order to give an order-of-magnitude estimation that takes into account all existing motif lengths and structures and their abundances. Requiring one motif-discriminating position maximizes the number of potential TFBS motifs to over 9700 (Fig 1B, black circles and right Y axis). The lower value for two or more motif-discriminating positions is due to higher non-overlap requirements. On the other hand, the value for zero motif-discriminating positions is lower than for at least one motif-discriminating position. This is the case because the condition of zero motif-discriminating positions acts on all positions of the motif at once (motifs in the set have common letters at all positions), while the condition of one motif-discriminating position restricts only one position at a time (motifs in the set are separated by one position). As a consequence, the overlap imposed by “zero motif-discriminating positions” is more restrictive than the non-overlap imposed by “one or more motif-discriminating position”. It is interesting to compare bars and circles of Fig 1B. On one hand, natural TFBS motif pairs are most often separated in sequence space by a single motif-discriminating position. On the other hand, this relatively low level of sequence specificity maximizes the number of potential TFBS motifs that can coexist while fulfilling the specificity requirement.

### Role of alphabet expansion

Current genome sequences frequently only inform the four canonical bases, and it is often forgotten that base modifications are varied and frequent [15] and can influence transcription factor binding [20, 21]. Regardless of the frequency of such modifications, an expanded DNA alphabet could potentially increase the number of potential TFBS motifs. An expanded DNA alphabet could be achieved by including new or modified bases using synthetic biology, while not the same as new bases, modified bases increase the capacity of DNA to code for TFBS motifs [20, 21] (section). We calculate the number of SwissRegulon Fantom5-like TFBS motifs that can potentially coexist in nature for expanded DNA alphabets including up to 10 different bases. Example calculations for the Arx TFBS motif are shown in the S1 Text. Fig 1C shows the median number of potential TFBS motifs as a function of alphabet size for 0 to 4 motif-discriminating positions. Increasing the alphabet size from 4 to 10 increases the number of potential TFBS motifs by several orders of magnitude for all specificity requirements tested. When we consider an effective alphabet size of 10 letters, the increase relative to an alphabet of four letters is highest at over 9500-fold for one motif-discriminating position (S4 Fig). This effect decreases sharply with increasing motif specificity, becoming lower than ten-fold for 9 or more motif-discriminating positions. This is notable since a single motif-discriminating position is the most frequent separation in sequence space between naturally occurring TFBS motifs (Fig 1B).

### Sequence space occupancy

A TFBS motif of length n is a subset of the sequence space of all possible 4^*n*^ DNA subsequences. We calculate the size of the sequence space determined by the regular expression for each SwissRegulon Fantom5 TFBS motif (section). S5 Fig shows that 82% of the TFBS motifs in our database potentially include between 1 and 10000 DNA subsequences, with over 50% of them including between 10 and 1000 DNA subsequences. We use this result and the corresponding maximum number of coexisting motifs to calculate the potential occupancy of sequence space for 1 to 10 motif-discriminating positions, i.e., the fraction of DNA subsequences of length n that are an instance of a motif if the maximum number of coexisting motifs is realized (section). The calculated values span several orders of magnitude (S6 Fig). As done for the number of potential motifs, Fig 1D reports the median of the distribution. For a single motif-discriminating position, a maximally large set of TFBS motifs occupies all the sequence space of length n: all possible DNA subsequences belong to a potential TFBS motif. The potential occupancy of sequence space drops steeply for two or more motif-discriminating positions. The commonest numbers of motif-discriminating positions (Fig 1B) maximize the potential occupancy of sequence space by the resulting TFBS motifs (Fig 1D). For a single motif-discriminating position, the potential occupancy of sequence space is 100% regardless of alphabet size (S7 Fig). For two or more motif-discriminating positions, the potential occupancy of sequence space is lower than one for an alphabet size of 4 and decreases further as alphabet size increases. For two or more motif-discriminating positions, increasing alphabet size leads to a trade-off between increasing the number of potential TFBS motifs (Fig 1C) and decreasing the potential occupancy of sequence space (S7 Fig).

## Discussion

The observed sequence specificity for human transcription factors (Fig 1A and 1B, bars) not only avoids most crosstalk between them but may also allow the simultaneous activity of several thousand TFBS motifs (Fig 1B, dots) that maximizes sequence space usage (Fig 1D). Increasing the DNA alphabet size would allow for an even larger number of TFBS motifs (Fig 1C). The results in (Fig 1C and 1D) are valid for any set of TFBS motifs, while the results in (Fig 1A and 1B) may vary to some degree as additional TFBS are described.

Studies of TFBS specificity usually look for similarities between the DNA binding preferences of transcription factors [25]. The main result is that DNA binding domains with similar protein sequences bind to similar sets of DNA subsequences [25]. On the other hand, we focus on quantifying in an intuitive and comprehensive manner the differences in specificity between human TFBS, which according to our definition are significant and widespread. Our finding that most human TFBS are separated in sequence space to some degree does not contradict the fact that many of them are similar to some degree. Let us consider two TFBS motifs of length 10. The two corresponding regular expressions are identical in the first nine positions and different only in position 10. The first motif allows A and C at position 10, while the second motif allows G and T at position 10. On one hand, the two motifs are similar since nine out of ten positions allow the same letters. On the other hand, no DNA subsequence can match both two regular expressions and the two motifs are separated in sequence space by one motif-discriminating position. In other words, a full understanding of TFBS motif specificity requires quantitative definitions for both motif similarity and motif separation in sequence space.

Regular expressions divide DNA subsequences into sites and non-sites, in parallel with the specific and non-specific modes of protein-DNA binding [26], but do not take into account affinity and transcription factor concentration. As a consequence, our model can describe how TFBS motifs make use of the available sequence space but does not attempt to describe the dynamics of transcription factor activity. It may be interesting to investigate the separation of TFBS in sequence space for other eukaryotic and prokaryotic organisms and in relation to the information content of the TFBS motif [10]. Since TFBS specificity is generally well conserved [25], we expect to find similar results in other species.

TFBS motifs from the SwissRegulon Fantom5 collection are commonly ten base pairs long, which corresponds to a space of ∼ 10^6^ DNA subsequences. Our theory, together with the observed sequence specificity, predicts that this sequence space can be organized into a maximum of ∼ 9.7 ⋅ 10^3^ TFBS motifs, separated by a single motif-discriminating position. In turn, coding theory [13] predicts a maximum of ∼ 4.5 ⋅ 10^3^ minimally overlapping TFBS motifs of length 10. A similar maximum of ∼ 1.6 ⋅ 10^4^ TFBS motifs can be obtained within the sphere packing approach of [14] and a minimal Hamming distance of 4 mutations between DNA subsequences belonging to different motifs. We find it reassuring that three different specificity-focused theories lead to estimates for the maximum number of TFBS motifs that are in the same order of magnitude. The actual upper bound for the number of TFBS motifs may be lower than 9700 due to phenomena not included in the theory. For example, the molecular interactions mediating protein-DNA interactions [6] may prevent some DNA subsequences from becoming actual TFBS motifs and a need for mutational robustness [27] may further constrain the maximal number of TFBS motifs. Also, binding of palindromic sequences by transcription factor dimers might cut the maximum number of TFBS up to 50%. This is because the binding sites of the two monomers would appear in our model as different TFBS motifs with regular expressions that are the reverse complement of each other, while actually being only one TFBS motif.

The symmetry in our equations implies that if a genome operated at or close the theoretical limit for the number of TFBS, the resulting base frequencies would all be 25% each. Thus, the maximum number of TFBS given by our calculation can be reached only in a genome whose TFBS have an overall GC content of 50%. This figure seems reasonable for the human genome, where the GC content is close to 41%. A quantitative assessment of this effect would require the deduction of additional theorems and will be addressed in future work. Similarly, if modified bases were present and the number of TFBS was close to the theoretical limit, the resulting frequencies of modified bases would be of the same magnitude as the frequencies of unmodified bases. Tackling this point would require the application of sequencing techniques sensitive to multiple modified bases at the genomic scale.

There are 2604 predicted DNA-binding proteins in the human proteome [7]. Since most of the 684 known human TFBS [12] are significantly separated in sequence space, we suggest that a significant number of human TFBS as defined in this work remain uncharacterized. This is compatible with the observation of conserved DNA subsequences of unknown function [28]. There is a second gap, between the 2604 predicted DNA-binding human proteins [7] and the predicted maximum number of ∼ 9.7 ⋅ 10^3^ coexisting TFBS motifs. This difference may be explained in terms of never born TFBS, which are physically possible but do not occur at present in nature due to incomplete exploration of the TFBS coding space during evolution [29].

Our theory is in principle valid for any set of molecules recognizing stretches of a linear polymer, regardless of the interacting partners. The overall picture for TFBS motifs is similar to our previous results for protein-protein interactions mediated by linear motifs [5]. In that case, the observed sequence specificity also maximizes the potential number of motifs up to around ten thousand. The main differences are that increasing the DNA alphabet size has a much larger effect than increasing the protein alphabet size and that sequence space usage is much larger for TFBS motifs than for protein linear motifs at the same level of specificity [5]. These differences arise from both alphabet size and the motif regular expressions, i.e., from the physicochemical basis of protein-protein versus protein-DNA complex formation [6].

The observation of 684 different TFBS motifs [12] and 2604 predicted DNA-binding proteins [7] suggests that encoding the binding sites for human transcription factors takes up 7 to 27% of the DNA subsequences of lengths 5 to 20. Other genome subsequences of functional significance, such as coding sequences or splicing sites also make use of the DNA sequence space. Because of this, our estimations for the potential number of coexisting TFBS motifs and for potential sequence space usage should be regarded as upper limits. It should also be considered that a given region of the genome may simultaneously code for different molecular activities. For example, the genetic code is nearly optimal for allowing additional information within protein-coding DNA subsequences [22].

Our results may aid the design of transcription factors at two different scales. At the molecular scale, the finding that naturally occurring human TFBS motifs are commonly separated in sequence space by one to three motif-discriminating positions may guide the design of new specific DNA binding proteins, be it TALEN, Zinc-finger, CAS9 or others. A specific DNA binding protein designed to function in a human cell should in principle have a low level of crosstalk with incumbent transcription factors, i.e., its binding site should be separated from most (if not all) other transcription factor binding sites by at least one motif-discriminating position. This is a well-defined design requirement that could be incorporated in current algorithms for the design of specific DNA binding proteins. At the network scale, the finding that the observed TFBS sequence specificity may also allow the coding a gene regulatory network with up to ten thousand TFBS motifs suggests that the human transcription factor binding site repertoire has not reached its maximum size and may be significantly enlarged through engineering [7, 30]. The use of an expanded DNA alphabet with modified bases may assist both scales of design.

## Supporting information

S1 Fig Example calculation for the number of motif-discriminating positions for two TFBS motifs (Arid3B and Arx). The two possible alignments are shown. All nine positions in the first alignment present at least one matching symbol. Thus, there is at least one DNA subsequence matching both regular expressions and the number of motif-discriminating positions for this alignment is 0. For the second alignment, seven positions present at least one matching symbol, while there is no overlap at positions 5 and 7. Thus, the number of motif-discriminating positions for this alignment is 2. The minimal number of motif-discriminating positions across the two possible alignments is zero. We take this number of motif-discriminating positions as a lower limit for the separation in sequence space between these two TFBS motifs.
(TIFF)

S2 Fig Separation in sequence space between TFBS motifs in the SwissRegulon Fantom5 collection that are not separated in sequence space by a motif-discriminating position. The X axis is the fraction of DNA subsequences matching any of the two regular expressions that match only one of them (i.e., 1 minus the Jaccard similarity index).
(TIFF)

S3 Fig Number of potential TFBS motifs as deduced from the SwissRegulon Fantom5 collection. Cumulative distribution function of the number of potential TFBS motifs for different numbers of motif-discriminating positions. Red: 0 positions. Black: 1 position. Dark green: 2 positions. Blue: 3 positions. Orange: 4 positions. Brown: 5 positions. Purple: 6 positions. Pink: 7 positions. Cyan: 8 positions. Magenta: 9 positions. Light green: 10 positions.
(TIFF)

S4 Fig Quotient of the number of potential TFBS motifs for alphabet sizes of 10 and 4, as a function of the number of motif-discriminating positions. (TIFF)

S5 Fig Histogram for the number of potential unique sequence instances belonging to a SwissRegulon Fantom5 TFBS motif, calculated from the corresponding regular expression. (TIFF)

S6 Fig Cumulative distribution function of the potential occupancy of the protein sequence space by TFBS motifs for different numbers of motif-discriminating positions. Black: 1 position. Green: 2 positions. Blue: 3 positions. Orange: 4 positions. Brown: 5 positions. Purple: 6 positions. Pink: 7 positions. Cyan: 8 positions. Magenta: 9 positions. Light green: 10 positions.
(TIFF)

S7 Fig Potential occupancy of the protein sequence space by TFBS motifs for different numbers of motif-discriminating positions, as a function of alphabet size. Black: 1 position. Red: 2 positions. Blue: 3 positions. Green: 4 positions.
(TIFF)

S1 Text Example calculations. (DOCX)

S1 File All TFBS regular expressions and data used in the figures. (ZIP)

## References

[1] Govindarajan S, Recabarren R, Goldstein Richard A. Estimating the total number of protein folds. Proteins: Structure, Function, and Bioinformatics. 1999;35(4):408–414. doi: 10.1002/(SICI)1097-0134(19990601)35:4<408::AID-PROT4>3.0.CO;2-A 10382668

[2] Wolf Yuri I, Grishin Nick V, Koonin Eugene V. Estimating the number of protein folds and families from complete genome data. Journal of molecular biology. 2000;299:897–905. doi: 10.1006/jmbi.2000.3786 10843846

[3] Aloy P, Russell RB. Ten thousand interactions for the molecular biologist. Nature biotechnology. 2004;22:1317–1321. doi: 10.1038/nbt1018 15470473

[4] Tompa P, Davey N, Gibson T, Babu M. A million peptide motifs for the molecular biologist. Mol Cell. 2014;55(2):161–169. doi: 10.1016/j.molcel.2014.05.032 25038412

[5] Bulavka D, Aptekmann AA, Méndez NA, Krick T, Sánchez IE. Thousands of protein linear motif classes may still be undiscovered. PLoS ONE. 2021;5(16):e0248841. doi: 10.1371/journal.pone.0248841 33939703 PMC8092775

[6] Rohs R, Jin X, West SM, Joshi R, Honig B, Mann RS. Origins of specificity in protein-DNA recognition. Annual review of biochemistry. 2010;79:233–269. doi: 10.1146/annurev-biochem-060408-091030 20334529 PMC3285485

[7] Babu MM, Luscombe NM, Aravind L, Gerstein M, Teichmann SA. Structure and evolution of transcriptional regulatory networks. Current opinion in structural biology. 2004;14(3):283–291. doi: 10.1016/j.sbi.2004.05.004 15193307

[8] Jolma A, Yan J, Whitington T, Toivonen J, Nitta K, Rastas P, et al. DNA-Binding Specificities of Human Transcription Factors. Cell. 2013;152(1-2):327–339. doi: 10.1016/j.cell.2012.12.009 23332764

[9] Friedlander T, Prizak R, Guet CC, Barton NH, Tkačik G. Intrinsic limits to gene regulation by global crosstalk. Nature Communications. 2016;7:1–12. doi: 10.1038/ncomms12307 27489144 PMC4976215

[10] Schneider TD, Stephens RM. Sequence logos: a new way to display consensus sequences. Nucleic acids research. 1990;18(20):6097–6100. doi: 10.1093/nar/18.20.6097 2172928 PMC332411

[11] Pachkov M, Erb I, Molina N, Van Nimwegen E. SwissRegulon: a database of genome-wide annotations of regulatory sites. Nucleic acids research. 2006;35(suppl_1):D127–D131. doi: 10.1093/nar/gkl857 17130146 PMC1716717

[12] Pachkov M, Balwierz PJ, Arnold P, Ozonov E, Van Nimwegen E. SwissRegulon, a database of genome-wide annotations of regulatory sites: recent updates. Nucleic acids research. 2012;41(D1):D214–D220. doi: 10.1093/nar/gks1145 23180783 PMC3531101

[13] Itzkovitz S, Tlusty T, Alon U. Coding limits on the number of transcription factors. BMC genomics. 2006;7(1):239. doi: 10.1186/1471-2164-7-239 16984633 PMC1590034

[14] Marathe A, Condon AE, Corn RM. On combinatorial DNA word design. Journal of Computational Biology. 2001;8(3):201–219. doi: 10.1089/10665270152530818 11535173

[15] Sood Ankur J, Viner C, Hoffman Michael M. DNAmod: the DNA modification database. Journal of cheminformatics. 2019;11(1):30. doi: 10.1186/s13321-019-0349-4 31016417 PMC6478773

[16] Ehrlich M, Wilson Geoffrey G, Kuo Kenneth C, Gehrke Charles W. N4-methylcytosine as a minor base in bacterial DNA. Journal of bacteriology. 1987;169(3):939–9432. doi: 10.1128/jb.169.3.939-943.1987 3029036 PMC211883

[17] Bachman M, Uribe-Lewis S, Yang X, Williams M, Murrell A, Balasubramanian S. 5-Hydroxymethylcytosine is a predominantly stable DNA modification. Nature chemistry. 2014;6(12):1049–1055. doi: 10.1038/nchem.2064 25411882 PMC4382525

[18] Neri F, Incarnato D, Krepelova A, Rapelli S, Anselmi F, Parlato C, et al. Single-Base Resolution Analysis of 5-Formyl and 5-Carboxyl Cytosine Reveals Promoter DNA Methylation Dynamics. Cell Reports. 2015;10(5):674–683. doi: 10.1016/j.celrep.2015.01.008 25660018

[19] Fu Y, Luo GZ, Chen K, Deng X, Yu M, Han D, et al. N6-methyldeoxyadenosine marks active transcription start sites in Chlamydomonas. Cell. 2015;161(4):879–892. doi: 10.1016/j.cell.2015.04.010 25936837 PMC4427561

[20] Fleming Aaron M, Ding Y, Burrows CJ. Oxidative DNA damage is epigenetic by regulating gene transcription via base excision repair. Proceedings of the National Academy of Sciences. 2017;114(10):2604–2609. doi: 10.1073/pnas.1619809114 PMC5347626 28143930

[21] Yin Y, Morgunova E, Jolma A, Kaasinen E, Sahu B, Khund-Sayeed S, et al. Impact of cytosine methylation on DNA binding specificities of human transcription factors. Science. 2017;356 (6337). doi: 10.1126/science.aaj2239 28473536 PMC8009048

[22] Itzkovitz S, Alon U. The genetic code is nearly optimal for allowing additional information within protein-coding sequences. Genome Res. 2007;17(4):405–412. doi: 10.1101/gr.5987307 17293451 PMC1832087

[23] Schneider T. Strong minor groove base conservation in sequence logos implies DNA distortion or base flipping during replication and transcription initiation. Nucleic acids research. 2001;29:4881–4891. doi: 10.1093/nar/29.23.4881 11726698 PMC96701

[24] Shannon CE. A mathematical theory of communication, Part I, Part II. Bell Syst Tech J. 1948;27:623–656. doi: 10.1002/j.1538-7305.1948.tb00917.x

[25] Weirauch MT, Yang A, Albu M, Cote AG, Montenegro-Montero A, Drewe P, et al. Determination and inference of eukaryotic transcription factor sequence specificity. Cell. 2014;158(6):1431–1443. doi: 10.1016/j.cell.2014.08.009 25215497 PMC4163041

[26] von Hippel PH, Berg OG. Facilitated target location in biological systems. J Biol Chem. 1989;264(2):675–678. doi: 10.1016/S0021-9258(19)84994-3 2642903

[27] Sengupta AM, Djordjevic M, Shraiman BI. Specificity and robustness in transcription control networks. Proceedings of the National Academy of Sciences. 2002;99(4):2072–2077. doi: 10.1073/pnas.022388499 11854503 PMC122321

[28] Bejerano G, Pheasant M, Makunin I, Stephen S, Kent WJ, Mattick JS, et al. Ultraconserved elements in the human genome. Science. 2004;304(5675):1321–1325. doi: 10.1126/science.1098119 15131266

[29] Szoniec G, Ogorzalek MJ. Entropy of never born protein sequences. Springerplus. 2013;2(1):200. doi: 10.1186/2193-1801-2-200 23750329 PMC3671101

[30] Verbič A, Praznik A, Jerala R. A guide to the design of synthetic gene networks in mammalian cells. FEBS J. 2020. 33289352 10.1111/febs.15652

