# Supplementary figures and images for: Transcription factor specificity limits the number of DNA-binding motifs
Source: PLoS One. 2022 Jan 28;17(1):e0263307. doi: 10.1371/journal.pone.0263307 (PMC8797260; doi:10.1371/journal.pone.0263307)

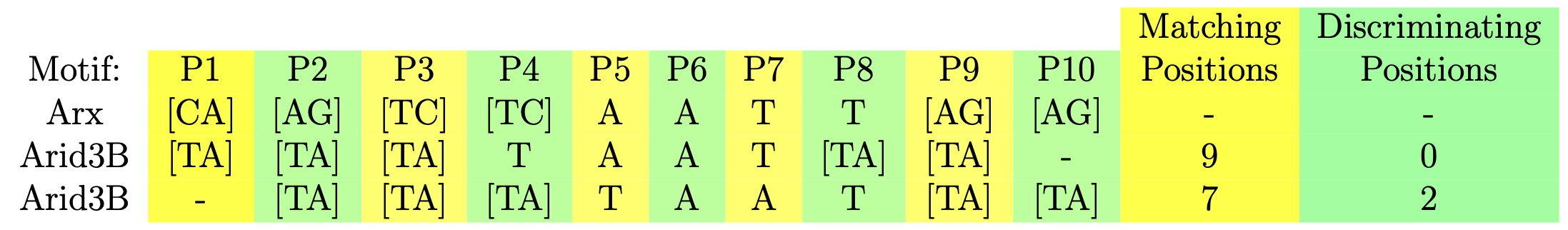

Supplement: S1 Fig — The two possible alignments are shown. All nine positions in the first alignment present at least one matching symbol. Thus, there is at least one DNA subsequence matching both regular expressions and the number of motif-discriminating positions for this alignment is 0. For the second alignment, seven positions present at least one matching symbol, while there is no overlap at positions 5 and 7. Thus, the number of motif-discriminating positions for this alignment is 2. The minimal number of motif-discriminating positions across the two possible alignments is zero. We take this number of motif-discriminating positions as a lower limit for the separation in sequence space between these two TFBS motifs. (TIFF) [file pone.0263307.s001.tiff]

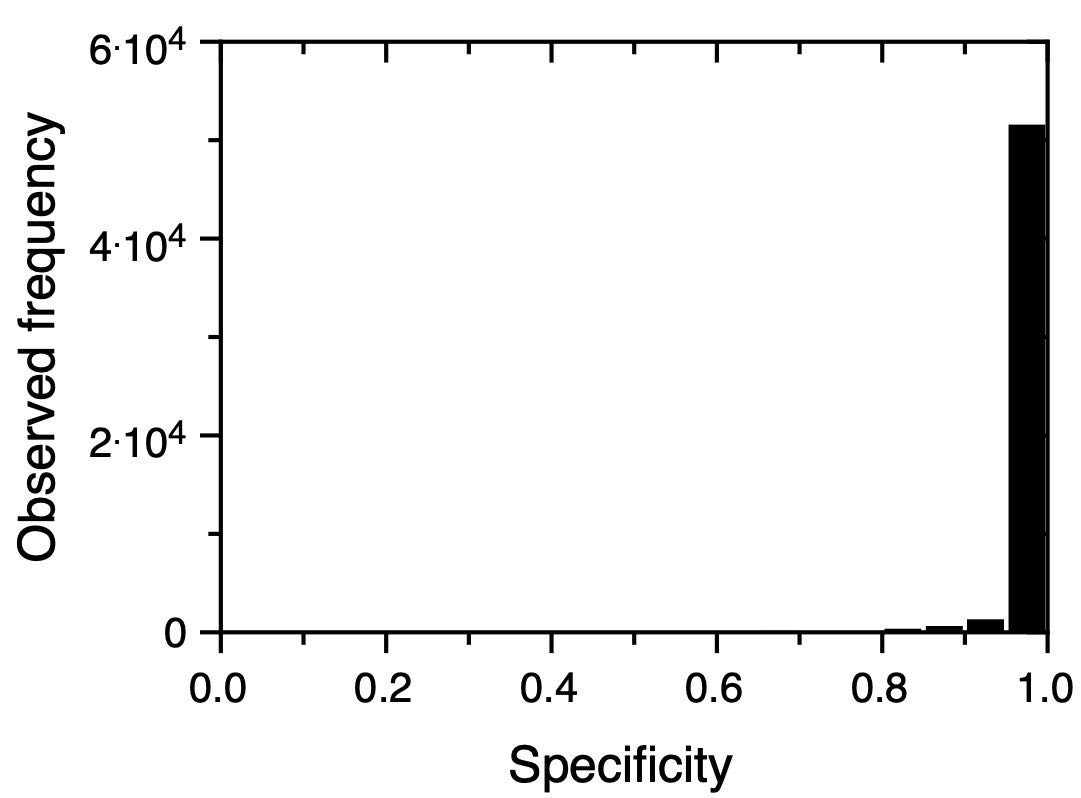

Supplement: S2 Fig — The X axis is the fraction of DNA subsequences matching any of the two regular expressions that match only one of them (i.e., 1 minus the Jaccard similarity index). (TIFF) [file pone.0263307.s002.tiff]

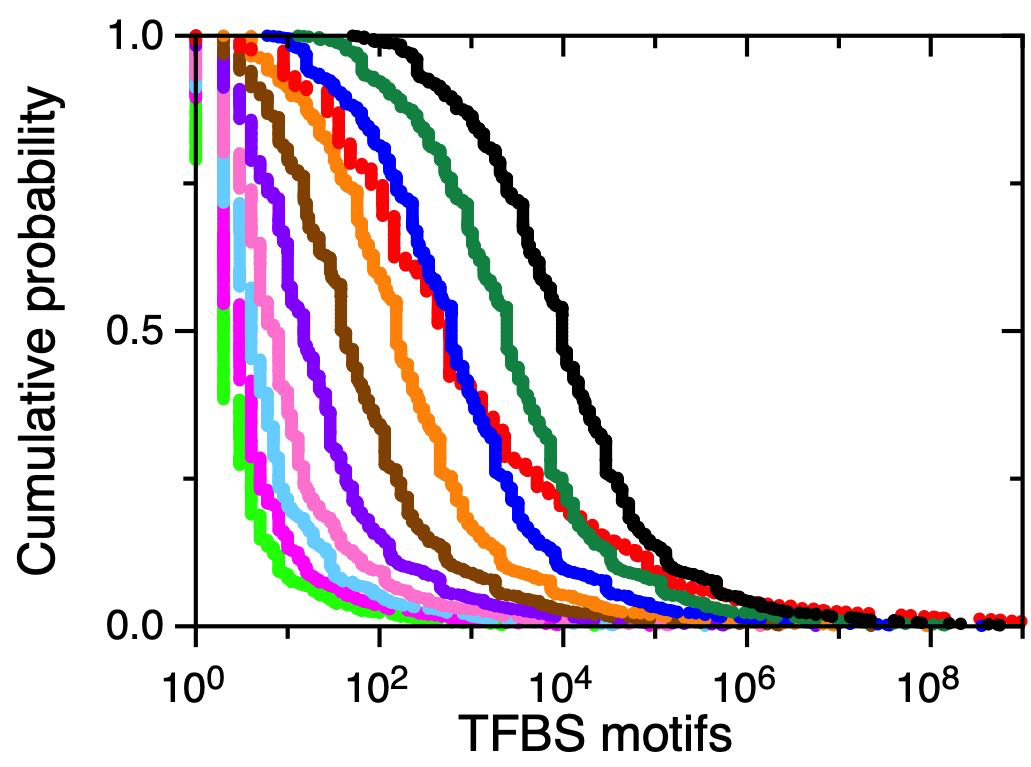

Supplement: S3 Fig — Cumulative distribution function of the number of potential TFBS motifs for different numbers of motif-discriminating positions. Red: 0 positions. Black: 1 position. Dark green: 2 positions. Blue: 3 positions. Orange: 4 positions. Brown: 5 positions. Purple: 6 positions. Pink: 7 positions. Cyan: 8 positions. Magenta: 9 positions. Light green: 10 positions. (TIFF) [file pone.0263307.s003.tiff]

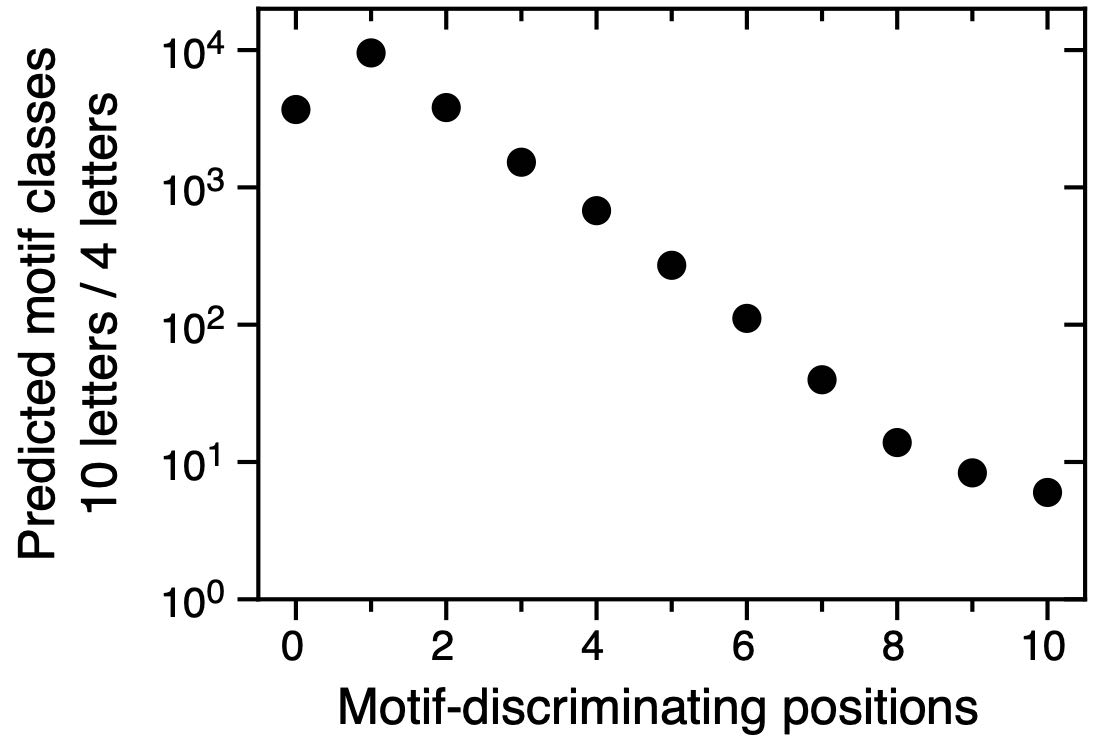

Supplement: S4 Fig — (TIFF) [file pone.0263307.s004.tiff]

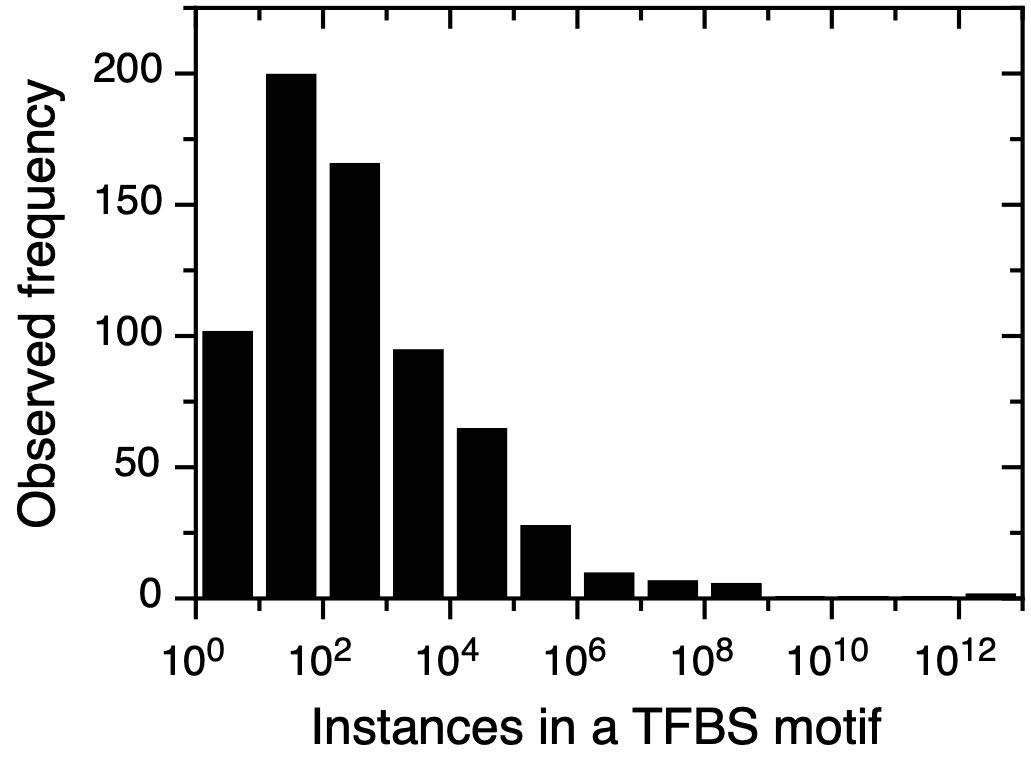

Supplement: S5 Fig — (TIFF) [file pone.0263307.s005.tiff]

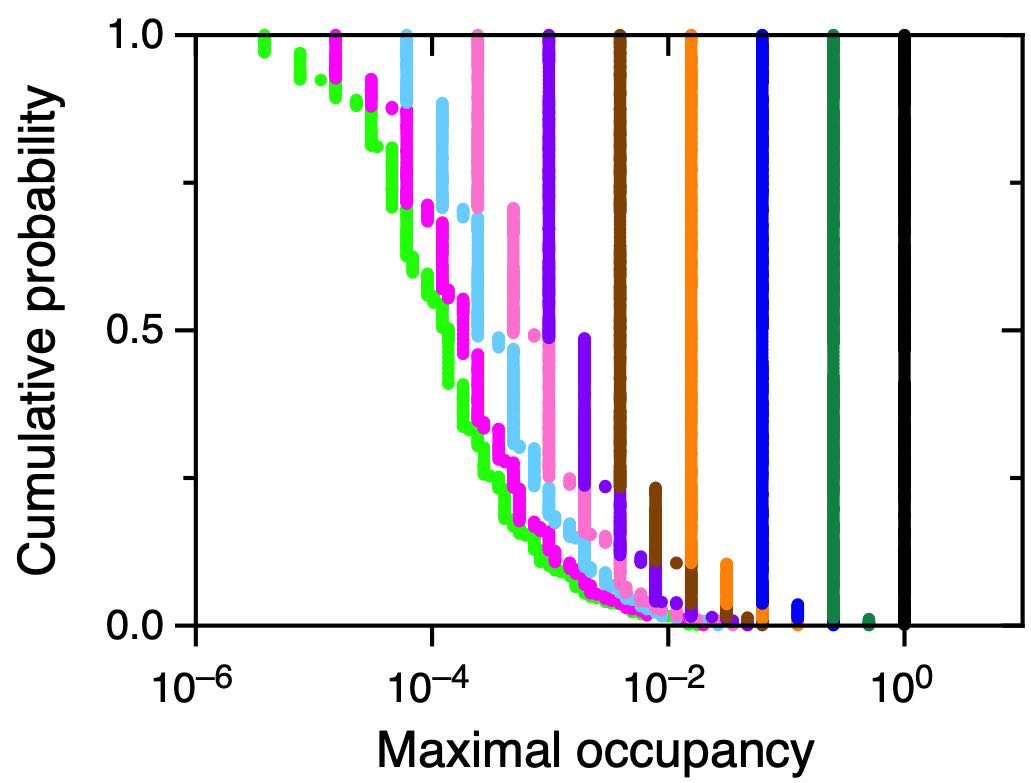

Supplement: S6 Fig — Black: 1 position. Green: 2 positions. Blue: 3 positions. Orange: 4 positions. Brown: 5 positions. Purple: 6 positions. Pink: 7 positions. Cyan: 8 positions. Magenta: 9 positions. Light green: 10 positions. (TIFF) [file pone.0263307.s006.tiff]

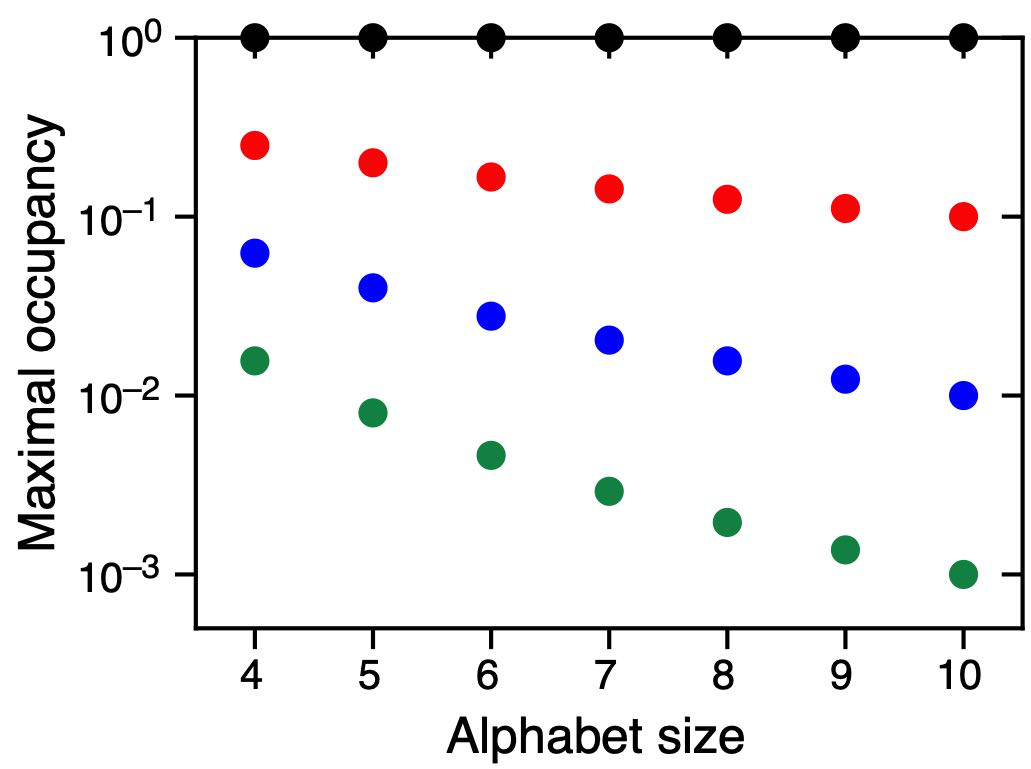

Supplement: S7 Fig — Black: 1 position. Red: 2 positions. Blue: 3 positions. Green: 4 positions. (TIFF) [file pone.0263307.s007.tiff]
