# Supplementary material for: Transcription factor specificity limits the number of DNA-binding motifs
Source: PLoS One. 2022 Jan 28;17(1):e0263307. doi: 10.1371/journal.pone.0263307 (PMC8797260; doi:10.1371/journal.pone.0263307)
Supplement: S1 Text — (DOCX) [file pone.0263307.s008.docx]

**Example calculations for the Arx TFBS motif**

*Regular expression*

The frequencies at the first position of the Arx TFBS motif matrix are:

A 0.265 C 0.275 G 0.173 T 0.287 (total base count is 9611).

From these frequencies, the Shannon entropy is 1.975 bits and the effective alphabet size (EAS) is 3.931. This number rounds to 4 allowed letters in this first position of the matrix. This is a flanking position that allows for all four bases and it is thus not taken into account for the Arx regular expression used in this work.

The frequencies at the second position of the Arx TFBS motif matrix are:

A 0.150 C 0.777 G 0.052 T 0.021 (total base count is 9611).

From these frequencies, the Shannon entropy is 1.031 bits and the effective alphabet size (EAS) is 2.043. This number rounds to 2 allowed letters in this second position of the matrix, which is the first one in our regular expression. The two most abundant bases are A and C, and the corresponding term in our regular expression is “[AC]”.

The frequencies at the sixth position of the Arx TFBS motif matrix are:

A 0.966 C 0.009 G 0.018 T 0.006 (total base count is 9611).

From these frequencies, the Shannon entropy is 0.263 bits and the effective alphabet size (EAS) is 1.200. This number rounds to 1 allowed letter in this sixth position of the matrix, which is the fifth one in our regular expression. The most abundant base is A, and the corresponding term in our regular expression is “A”.

Following this procedure for all positions in the original matrix leads to calculation of the regular expression [CA][AG][TC][TC]AATT[AG][AG] for the Arx TFBS motif.

*Number of potential transcription factor binding site motifs*

Let us perform the calculation for the regular expression [CA][AG][TC][TC]AATT[AG][AG] of the Arx TFBS motif. Its length is 10 and its structure is (2,2,2,2,1,1,1,1,2,2). If we impose that all pairs of TFBS motifs present at least one motif-discriminating position, k = 1, the number of potential TFBS motifs that can coexist is (4/2)*(4/2)*(4/2)*(4/2)*4*4*4*4*(4/2)*(4/2) = 16384 according to Equation 5 of the main text (see table for other values of k).

| **k** | 0 | 1 | 2 | 3 | 4 | 5 |
| --- | --- | --- | --- | --- | --- | --- |
| **Predicted TFBS motifs** | 729 | 16384 | 4096 | 1024 | 256 | 64 |

We note that these figures are independent of the order of the e_i_, so that calculations for regular expressions with the structures (2,2,2,2,1,1,1,1,2,2) and (2,2,2,2,2,2,1,1,1,1) yield the same results.

*Role of base modifications*

Next, we examined the effect of increasing alphabet size on the number of potential TFBS motifs for the Arx TFBS motif. In the case of a single motif-discriminating position, the number of potential TFBS motifs is given by Equation 4 in section 4.3 of the main text. For an alphabet size of 4, the number of potential TFBS motifs of this structure is (4/2)*(4/2)*(4/2)*(4/2)*4*4*4*4*(4/2)*(4/2) = 16384. This number goes up to (10/2)*(10/2)*(10/2)*(10/2)*10*10*10*10*(10/2)*(10/2) = 156250000 for an alphabet size of 10.

*Sequence space occupancy*

Let us calculate the occupancy of sequence space for the Arx TFBS motif. This is a TFBS motif of length 10, where 6 positions admit 2 bases and 4 positions admit 1 base. The product of the permitted bases per position shows how many instances could belong to any single TFBS motif of this structure, that is, 2*2*2*2*1*1*1*1*2*2 = 64. On the other hand, for a length of 10 the number of all possible DNA subsequences is 4^10^ = 1048576. The occupancy for this TFBS motif then is the ratio between these two numbers, 64/1048576 = 0.00006103515625.

Next, we can apply Equation 7 of the main text to calculate the total occupancy of sequence space for all possible TFBS motifs with structure (2,2,2,2,1,1,1,1,2,2). That is, what fraction of the DNA sequence space would all the instances of all the possible TFBS motifs occupy, for a given structure and k. For this TFBS motif structure and k=1, the total number of possible TFBS motifs is 16384 and the total occupancy of sequence space is 16384*0.00006103515625 = 1. Note that we can perform the calculation in this relatively intuitive way only for k = 1 or higher.
